# High Prevalence of Probable Sarcopenia and Its Associations with Nutrition, Cognitive, and Physical Function in Hospitalized Patients with Alzheimer’s Clinical Syndrome: A Cross-Sectional Study

**DOI:** 10.3390/nu18020347

**Published:** 2026-01-21

**Authors:** Vesna Simič, Nina Mohorko, Polona Rus Prelog

**Affiliations:** 1 Faculty of Health Science, University of Primorska, Polje 42, 6310 Izola, Slovenia; nina.mohorko@upr.si; 2 Centre for Clinical Psychiatry, University Psychiatric Clinic Ljubljana, Chengdujska 45, 1260 Ljubljana, Slovenia; polona.rus@psih-klinika.si; 3 Faculty of Medicine, University of Ljubljana, Vrazov Trg 2, 1000 Ljubljana, Slovenia

**Keywords:** sarcopenia, muscle strength, Alzheimer’s disease, cognitive function, gait speed, nutrition risk

## Abstract

Background: Probable sarcopenia, indicated by low handgrip strength, is a prevalent condition among hospitalized older adults and may reflect broader functional and nutritional decline. Methods: We examined differences in nutritional, functional, and cognitive status between Alzheimer’s clinical syndrome (ACS) patients with probable sarcopenia and those without sarcopenia. A cross-sectional analysis was conducted on 194 hospitalized older adults with ACS. Probable sarcopenia was defined using European Working Group on Sarcopenia in Older People (EWGSOP2) handgrip strength thresholds. Results: Patients with probable sarcopenia (*n* = 137) had significantly lower Mini-Mental State Examination (MMSE) scores, Geriatric Nutritional Risk Index (GNRI), albumin, hemoglobin, and gait speed compared to those without. After age and sex adjustment, MMSE (*p* = 0.023), GNRI (*p* = 0.002), hemoglobin (*p* = 0.022), albumin (*p* = 0.003), and gait speed (*p* < 0.001) remained significantly different. In the sex- and age-adjusted multivariable model (adjusted R^2^ = 0.442), higher nutritional risk (β = 0.26, *p* = < 0.001), lower MMSE scores (β = 0.17, *p* = 0.029), polypharmacy (β = −4.20, *p* = 0.002), and slower gait speed (β = 4.12, *p* = 0.010) were associated with reduced handgrip strength. In the multivariable binary logistic regression model (adjusted for age and sex), moderate or high nutritional risk and slow gait speed emerged as independent predictors of probable sarcopenia, with OR 5.14 (95% CI 1.34–19.75; *p* = 0.017) and OR 3.13 (95% CI 1.30–7.52; *p* = 0.011), respectively. Conclusions: Probable sarcopenia in hospitalized older adults with ACS is highly prevalent and is associated with higher nutritional risk, poorer cognitive and physical function, and polypharmacy; its early recognition may help to guide more targeted nutritional and functional interventions.

## 1. Introduction

As the global population ages, the number of older adults with age-related diseases, frequently experiencing several conditions at the same time, is rising sharply [1], leading to significant medical and socioeconomic challenges. Among the leading contributors to disabilities are cognitive impairment and sarcopenia, now representing two of the four new modern “giants of geriatrics”, together with frailty and anorexia of aging [2]. Dementia is considered the most advanced and debilitating form of cognitive impairment. Alzheimer’s disease (AD) is the most common cause of dementia, accounting for an estimated 60% to 80% of cases [3]. It is a progressive and fatal neurodegenerative disorder, marked by declining cognitive and memory functions, with the emergence of various neuropsychiatric symptoms, behavioral disturbances, and a gradual loss of ability to perform daily activities [4]. In contemporary practice, key biomarkers of amyloid, tau, and neurodegeneration play a central role in disease identification [5]; in the absence of biomarker confirmation, the clinical picture consistent with AD is commonly referred to as Alzheimer’s clinical syndrome (ACS).

Sarcopenia is defined as a progressive and generalized skeletal muscle disorder characterized by a loss of muscle mass and muscle function [6] and is consistently and strongly associated with several adverse outcomes, including increased risk of mortality, reduced health-related quality of life, and increased risk of falls and fractures [7]. Sarcopenia has a multifactorial etiology, which is not completely understood, but some risk factors have been identified: changes in protein metabolism and inadequate diet, chronic inflammation, hormonal alterations, loss of alpha motor neurons, and reduced physical activity [8]. Importantly, prevention and treatment of sarcopenia are possible and could be done primarily through nutritional and exercise intervention, with the emerging interest in pharmacological strategies [9,10]. Despite these advances, sarcopenia remains underdiagnosed and undertreated [11], in part due to the lack of universally accepted diagnostic criteria in the past. Sarcopenia is common in patients with AD, and according to the recently published systematic review and meta-analysis, it was found in 31.2% and 41.9% of patients with mild and moderate AD, respectively, which is significantly higher than the prevalence reported in the general elderly population [12]. The highest prevalence of 55,7% was found in AD patients older than 80 years [12].

Growing evidence indicates that sarcopenia and cognitive decline frequently coexist in older adults and may share common biological mechanisms mediated through the muscle–brain axis. Skeletal muscle is increasingly recognized as an active endocrine organ capable of influencing brain function via myokines, mitochondrial signaling, and inflammatory pathways, thereby linking age-related declines in muscle strength and function with neurocognitive impairment [13]. In parallel, exercise-induced improvements in mitochondrial function within skeletal muscle have been shown to exert neuroprotective effects, supporting a mechanistic connection between physical activity, muscle metabolism, and reduced risk of neurodegenerative diseases, including Alzheimer’s disease [14]. More recently, the concept of a broader gut–muscle–brain axis has emerged, highlighting the multidirectional crosstalk among skeletal muscle, gut microbiota, and the central nervous system. Physical activity and nutritional factors can modulate gut microbiota composition, short-chain fatty acid production, inflammation, and muscle metabolism, with downstream effects on cognitive function and neurodegeneration [15]. Meta-analyses consistently confirmed that sarcopenia was associated with cognitive impairment [16,17,18]. The interplay between sarcopenia and AD is especially concerning, as it may accelerate the progression of both conditions, creating a vicious cycle of declining physical and cognitive function. Therefore, early identification and management of sarcopenia as a potentially modifiable condition are essential, as they may prevent further functional decline.

According to the revised European Working Group on Sarcopenia in Older People criteria (EWGSOP2), sarcopenia is defined through a stepwise diagnostic process. Muscle strength is first assessed, typically using handgrip strength; if muscle strength is low, sarcopenia is confirmed by evaluating muscle mass quantity or quality, and its severity is then determined by assessing physical performance, for example, by measuring gait speed [6]. EWGSOP2 introduced the concept of “probable sarcopenia,” which is based on low muscle strength alone, primarily handgrip strength (HGS) [6]. In this 2019 revision, the low HGS replaced low muscle mass as the primary diagnostic criterion for sarcopenia, as it is a more sensitive predictor of negative outcomes such as longer hospital stays, increased functional limitations, poor health-related quality of life, and death [6]. HGS is a simple, quick, and inexpensive measure not only of muscle function but also an indicator of overall health as well [19], and it can be used in both clinical practice and research. Sex and age are the two primary determinants of handgrip strength, with sex accounting for the largest share of overall variability [20].

Research on sarcopenia and its determinants—handgrip strength and gait speed—in patients with AD is very limited, particularly in advanced stages of the disease, as patients with severe cognitive decline are often not included in research [12,21,22], which does not accurately reflect real-life clinical practice. Given the high prevalence of sarcopenia among hospitalized AD elderly patients, identifying the key factors related to HGS, the main sarcopenia determinant, would have important clinical implications. The aim of this study was to investigate the prevalence of probable sarcopenia among hospitalized patients with all stages of ACS. Furthermore, our goal was to examine differences in nutritional risk and cognitive and physical function between patients with probable sarcopenia and those without sarcopenia, and to identify associations and independent predictors of probable sarcopenia in this vulnerable population.

## 2. Materials and Methods

### 2.1. Study Design and Participants

**Figure 1:**
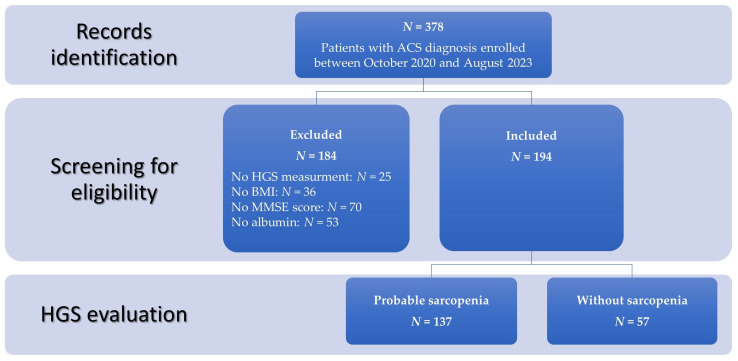
Study flow chart. ACS, Alzheimer’s clinical syndrome; HGS, handgrip strength; BMI, body mass index; MMSE, Mini-Mental State Examination.

This retrospective cross-sectional study included 378 clinical records of ACS patients (131 men, 247 women) who were admitted to the geriatric psychiatry ward from October 2020 to August 2023 with a diagnosis of ACS accompanied by worsening psychiatric symptoms. The records were screened for eligibility: records of patients without HGS measurement, no body weight or height data, no Mini-Mental State Examination (MMSE) score, or no albumin (needed for Geriatric Nutritional Risk Index (GNRI) calculation) were excluded from the study (Figure 1). For each patient, the diagnosis of ACS was established by an experienced psychiatrist in accordance with the 10th edition of the International Classification of Diseases (ICD-10) [23]. Diagnoses were based on a comprehensive clinical evaluation, including a detailed medical history, reports from caregivers and the patient, assessment of mental status, physical and neurological examination, standardized neuropsychological testing, and structural neuroimaging (magnetic resonance imaging or computed tomography). When clinically indicated, additional biomarker investigations were performed, including cerebrospinal fluid analysis and/or positron emission tomography, following the National Institute on Aging–Alzheimer’s Association (NIA-AA) 2018 framework [24] for AD. Data collection took place between October 2020 and August 2023, prior to the publication of the revised 2024 NIA–AA criteria [5], and reflects routine clinical diagnostic practice in a geriatric psychiatry setting, where systematic biomarker confirmation was not universally available or required for diagnosis. Accordingly, and in alignment with the NIA–AA research framework [5], the study population is referred to as having Alzheimer’s clinical syndrome (ACS) throughout the manuscript when describing the cohort and the data derived from this study. The presence of comorbidities or severe cognitive decline was not an exclusion factor. Inpatients routinely underwent laboratory tests and assessment of physical functions, including HGS and gait speed. HGS and gait speed were assessed within the first 48–72 h after hospital admission, once patients were stable, without marked acute clinical deterioration, and able to follow simple instructions, with assistance if required, in order to minimize the confounding effects of acute illness on muscle strength assessment. Patient information, including their living environment prior to admission, age, weight, height, body mass index (BMI), MMSE score, laboratory blood/serum markers, physical function test results, medications, and diagnosis, was obtained from the clinic’s database. This study was approved by the Commission for Ethical Issues at the University Psychiatric Clinic Ljubljana (reference number KE-UPKL/2024-06). The study was performed in accordance with the Declaration of Helsinki and its subsequent amendments. As this study analyzed data obtained during routine clinical practice and involved no additional interventions or contact with patients, no specific consent for data processing was required. To ensure compliance with data protection standards, all collected data were anonymized prior to analysis.

### 2.2. Clinical Information

Demographic factors, including age, sex, and patients’ living environment prior to admission, were collected. Number of regularly used medications and polypharmacy, defined as the regular use of five or more medications at the same time [25], were assessed. We determined the prevalence of major chronic diseases of older adults using the age-adjusted Charlson comorbidity index (CCI). This index provides a simple and valid method of estimating the risk of death from comorbid disease for use in longitudinal studies. CCI is a global score (0 to 31 points) that assesses age and the presence of 16 different comorbidities (myocardial infarction, congestive heart failure, peripheral vascular disease, cerebrovascular accident/transient ischemic attacks, dementia, chronic obstructive pulmonary disease, connective tissue disease, peptic ulcer disease, liver disease, diabetes mellitus, hemiplegia, chronic kidney disease, solid tumor, leukemia, lymphoma, and AIDS) [26].

### 2.3. Cognitive Function

The severity of cognitive impairment was assessed using the Slovenian version of MMSE, which evaluates orientation, attention, memory, language, and visuospatial skills, with a maximum score of 30 points [27]. Patients with ACS were divided into mild ACS (MMSE score ≥ 19), moderate ACS (MMSE score 11–18 points), and severe ACS (MMSE score ≤ 10 points).

### 2.4. Nutrition-Related Variables

Body weight (to the nearest 0.1 kg) and height (to the nearest 0.1 cm) were measured according to the standard protocol, and BMI was calculated [28]. The venous blood samples of ACS patients were collected in a fasting state the morning after admission and then sent to the clinical laboratory. A variety of nutrition-related laboratory variables in blood/serum to assess the nutritional status of patients, including hemoglobin, fasting blood glucose, triglycerides, LDL, HDL, urea nitrogen, creatinine, albumin, folic acid, and vitamin B12, are routinely collected. Nutrition-related risk was determined using the GNRI, an objective screening tool developed to predict the risk of nutrition-related complications in older people [29]. The GNRI was calculated with the following formula:

GNRI = [1.489 × serum albumin (g/L)] + [41.7 × (present weight/ideal weight (kg))].(1)

The Lorentz formula was used to calculate ideal body weight according to the patients’ height and sex, as follows [30]:

height (cm) − 100 − {(height (cm) − 150)/4} for men and(2)

height (cm) − 100 − {(height (cm) − 150)/2.5} for women.(3)

When the present weight/ideal body weight ratio was ≥1, the ratio was set to 1. Patients were categorized according to the following threshold values: major risk (GNRI < 82), moderate risk (GNRI 82 to <92), low risk (GNRI 92 to ≤98), and no risk (GNRI > 98) [29].

### 2.5. Physical Function Related Variables

Patients underwent an HGS measurement administered by trained physiotherapists as a standardized clinical pathway for assessing physical functions in individuals with dementia. HGS was measured using the Saehan Hydraulic Hand Dynamometer, set to the second handle position. Standardized protocol, as described elsewhere, was followed [31]. During test administration, adjustments were made as needed, such as simplifying instructions or using practical demonstrations. To support patient understanding, repeated verbal prompts were often provided throughout the testing process. Breaks were incorporated when necessary to ensure comfort. To minimize fatigue, each test was performed only once, with no repetitions. Scores below 27 kg for men and 16 kg for women were used to identify probable sarcopenia using EWGSP2 recommended cut-offs [6]. Gait speed was assessed using the 6 m walking test. Patients were instructed to walk a 10 m corridor at their usual walking speed. To account for acceleration and deceleration, only the time taken to walk the middle 6 m was measured, excluding the first and last 2 m. A stopwatch was used to record the time. Gait speed below 0.8 m per second was classified as low and was associated with poor clinical outcomes [6].

### 2.6. Statistical Analysis

The data are presented as means ± standard deviations or as frequencies and percentages. Group differences were assessed using the Chi-square test, independent-samples *t*-test, Mann–Whitney U test, analysis of variance (ANOVA), and Kruskal–Wallis H test, as appropriate. The threshold for statistical significance was set at α < 0.05, and all analyses were carried out by IBM SPSS statistical software (version 29.0, IBM, Armonk, NY, USA). Assumptions of normality and homogeneity of variance were evaluated using Kolmogorov–Smirnov’s test and Levene’s test, respectively. Additionally, an analysis of covariance (ANCOVA) was conducted to control for age and sex. Assumptions for ANCOVA, including linearity and homogeneity of regression slopes, were also assessed and confirmed. Visual inspections of histograms and scatterplots were performed to further verify data distribution and identify the outliers. To explore associations and predictors of probable sarcopenia, age- and sex-adjusted multivariable linear regression and multivariable binary logistic regression models were applied.

## 3. Results

The study included 378 records of hospitalized elderly patients with ACS (34.7% women) with a mean age of 81.5 ± 7.9 years. A total of 194 records (51.3%) were included in the final analysis (56 men and 138 women; mean age 80.9 ± 7.6 years, 66–96 years old, with mean HGS 14.5 ± 8.4 kg). Based on the MMSE score, 38.7% patients had mild cognitive decline, 39.7% moderate, and 21.6% had severe cognitive decline. Among the 194 hospitalized patients with ACS included in this study, 137 (70.6%) met the criteria for probable sarcopenia based on EWGSOP2 criteria. The comparison between patients with and without probable sarcopenia revealed significant differences in demographic, nutritional, laboratory, and functional characteristics (Table 1). Patients with probable sarcopenia were significantly older than those without (82.3 ± 7.2 vs. 77.8 ± 7.5 years; *p* < 0.001) and had lower MMSE scores, indicating more pronounced cognitive impairment (15.3 ± 6.0 vs. 18.1 ± 6.7; *p* = 0.005). Nutritional risk was significantly higher, reflected by lower GNRI values (97.6 ± 8.2 vs. 102.6 ± 7.3; *p* < 0.001) and was more prevalent in the patients with probable sarcopenia (*p* = 0.006).

From a biochemical perspective, patients with probable sarcopenia had significantly lower serum albumin levels (38.4 ± 4.9 vs. 41.4 ± 4.3 g/L; *p* < 0.001), hemoglobin concentrations (127.7 ± 16.3 vs. 133.8 ± 11.7 g/L; *p* = 0.003), and HDL cholesterol (1.2 ± 0.3 vs. 1.3 ± 0.3 mmol/L, *p* = 0.015). Serum urea levels were significantly higher in the group with probable sarcopenia (7.8 ± 4.0 vs. 6.5 ± 3.3 mmol/L, *p* = 0.017). Patients with probable sarcopenia had a higher comorbidity burden, as measured by CCI (6.1 ± 1.6 vs. 5.1 ± 1.2; *p* < 0.001), indicating a more complex clinical profile (89.8% vs. 75.4%, *p* = 0.010). Additionally, polypharmacy (defined as >5 medications) was significantly more common in this group (89.8% vs. 75.4%; *p* = 0.010). Functionally, patients with probable sarcopenia had slower gait speed (0.5 ± 0.3 vs. 0.8 ± 0.3 m/s; *p* < 0.001), with 86.2% of them walking at ≤0.8 m/s compared with 61.4% in the patients without sarcopenia.

No statistically significant differences were observed between the groups in terms of BMI, prevalence of undernutrition based on BMI; presence of diabetes mellitus; serum levels of creatinine, glucose, folic acid, and vitamin B12; and total cholesterol levels.

ANCOVA analyses, adjusted for age and sex, confirmed the significance of differences in GNRI, albumin, hemoglobin, CCI, gait speed, and MMSE between groups, indicating that these associations are independent of age and sex.

In the multivariable linear regression model adjusted for age and sex, higher GNRI scores (β = 0.26, *p* = < 0.001), faster gait speed (β = 4.12, *p* = 0.010), and higher MMSE scores (β = 0.17, *p* = 0.029) were independently associated with greater handgrip strength, whereas polypharmacy (β = −4.20, *p* = 0.002) and female sex (β = −7.30, *p* = < 0.001) were associated with lower handgrip strength (Table 2). Hemoglobin, urea, vitamin B12, folate, comorbidity burden, and age were not independently associated with handgrip strength after adjustment.

In the multivariable binary logistic regression analysis adjusted for age and sex (Table 3), nutritional status and functional performance emerged as the strongest predictors of probable sarcopenia (Nagelkerke R^2^ = 0.330). Compared with participants without nutritional risk (GNRI > 98), those with moderate or high nutritional risk (GNRI < 92) had significantly higher odds of the outcome (OR 5.14, 95% CI 1.34–19.75; *p* = 0.017). Mild nutritional risk (GNRI 92–98) was not significantly associated with the outcome (OR 1.99, 95% CI 0.75–5.29; *p* = 0.170). A gait speed of ≤0.8 m/s was independently associated with increased odds (OR 3.13, 95% CI 1.30–7.52; *p* = 0.011). Age and polypharmacy did not reach statistical significance but showed a trend toward association with the outcome. No other clinical or laboratory variables were independently associated with the outcome after adjustment.

## 4. Discussion

The study evaluated the prevalence of probable sarcopenia and examined the differences in nutritional risk and cognitive and physical function among hospitalized patients across all stages of ACS with probable sarcopenia and without sarcopenia. Patients with probable sarcopenia demonstrated significantly worse cognitive performance, physical function, and higher nutritional risk based on malnutrition-related indicators. Lower GNRI, lower MMSE score, lower gait speed, and polypharmacy were associated with reduced handgrip strength and accounted for 44.2% of the variance in a multivariable linear regression model, adjusted for sex and age. In an age- and sex-adjusted multivariable binary logistic regression model, moderate or high nutritional risk and slow gait speed emerged as independent predictors of low handgrip strength.

The present study confirms a strong association between probable sarcopenia and several indicators of poor health status in hospitalized older adults with ACS. Our findings support the growing body of evidence suggesting that sarcopenia and cognitive impairment frequently coexist and may mutually exacerbate each other, knowing that both diseases have common underlying conditions such as reduced physical activity and dietary intake, inflammatory processes, oxidative stress, and hormonal changes [32]. Literature suggests that the relationship between sarcopenia and cognitive impairment is driven by the decline in HGS as the hallmark of sarcopenia, and not the muscle mass loss [32,33,34], suggesting low HGS might be an early non-cognitive feature of elderly patients with AD [21].

With over 70% of our ACS patients meeting the criteria for probable sarcopenia according to EWGSOP2 criteria, the prevalence was higher than described previously in two cross-sectional studies of 128 and 133 AD patients, where the frequency of probable sarcopenia was 54.7% and 53.4%, respectively [34,35]. Our higher prevalence of probable sarcopenia in ACS patients might be due to the older patients included in our study (80.9 ± 7.6 years vs. 76.56 ± 7.54 years and 76.33 ± 7.45 years) and the inclusion of patients with severe forms of disease, who were excluded from the aforementioned studies [34,35]. The frequency of probable sarcopenia observed in our study was also substantially higher than the 20–45% reported in the community-dwelling elderly population [36,37,38,39] and 25–44% in hospitalized patients with various diseases [40,41], underscoring the clinical relevance of low muscle strength in dementia care settings.

### 4.1. Cognitive Function and Muscle Strength

Our study found that cognitive decline, as measured by the MMSE, was steeper in patients with probable sarcopenia, even after adjustment for sex and age. MMSE was significantly associated with muscle strength, consistent with previous evidence linking cognitive decline to muscle mass and muscle function decline in the elderly with AD [18,21,34]. Over the past decade, growing attention has been given to the link between sarcopenia and cognitive impairment. A recent meta-analysis of 27 studies found that individuals with sarcopenia or probable sarcopenia were nearly twice as likely to experience cognitive decline compared to those without [42]. Notably, this strong association is consistent regardless of geographic region, study population, or the criteria used to define sarcopenia and cognitive impairment [18]. HGS, the main determinant of sarcopenia according to the EWGSOP2 criteria, is increasingly recognized not only as a marker of overall muscle strength but also as a predictive indicator of cognitive health, disability, morbidity, and mortality in older adults [20]. In AD patients, regardless of the stage of disease (early, mild, or moderate AD), lower HGS was observed than in patients with normal cognition [21].

Not only do higher MMSE scores predict better muscle strength, but there is also evidence of a bidirectional effect of HGS and cognitive function [43]. The bidirectional association thus suggests that preventing muscle strength decline is important not only for maintaining functionality but also for maintaining cognitive function or even slowing cognitive decline. Further, higher baseline handgrip strength has been identified as a protective factor against the development of AD [20,44].

The relationship between AD and low muscle strength is important for the clinical outcomes, as sarcopenia can exacerbate the current functional limitations in AD, leading to a more pronounced decline in the ability to perform daily activities and adding to the burden on caregivers [45].

### 4.2. Nutritional Risk and Muscle Strength

Older adults with AD have a high prevalence of both malnutrition and sarcopenia, with low handgrip strength serving as a hallmark of both conditions [6,46]. Malnutrition becomes more pronounced as AD progresses, with lower BMI; reduced protein, albumin, B12, and folic acid levels; and diminished body circumferences being common findings in this population [47]. Hence, these parameters are usually routinely monitored in clinical practice.

Our study confirmed higher nutritional risk in the probable sarcopenic group with different indicators of nutritional status, like GNRI, albumin, and hemoglobin, but not with B12 and folic acid. The latter two could be explained based on the frequent routine supplementation of these two vitamins in AD patients, which may confound the results. Albumin levels were significantly lower in patients with probable sarcopenia in both unadjusted and age- and sex-adjusted analyses, which may indicate a higher nutritional risk. However, previous studies have suggested that low albumin, while potentially reflecting undernutrition, may also be strongly influenced by systemic illness and inflammation rather than by direct nutritional causality [48]. Hemoglobin level, a diagnostic criterium for anemia, was also lower in patients with probable sarcopenia. Nutritional deficiencies—particularly of vitamin B12, folate, and iron—account for approximately one-third of anemia cases in older adults and have been linked to cognitive impairment [49]. While anemia can contribute to cognitive decline by reducing oxygen delivery to the brain as well as functional decline by limiting oxygen supply to the muscles, the mean hemoglobin concentrations in both groups lie within a range that is generally above thresholds associated with overt anemia and clinically manifest hypoxic symptoms, so any impact of mild reductions in hemoglobin on cognition and function should be interpreted with caution [50].

Although BMI did not differ significantly between the groups, nutritional risk, measured by GNRI, was significantly higher and more prevalent (unadjusted and sex- and age-adjusted) in patients with probable sarcopenia, reinforcing the idea that BMI alone is not a sensitive indicator of nutritional status or muscle health in older adults [6,51]. Particularly in our ACS group with 14.4% of patients having BMI > 30 kg/m^2^, sarcopenia might have been masked by preserved or even elevated fat mass (i.e., sarcopenic obesity), which further complicates the identification of at-risk individuals using traditional anthropometric indicators alone. GNRI is a nutritional screening index that was originally proposed to assess the nutrition-related risk for hospitalized elderly [29]. The GNRI is a simple and objective index, which, unlike other nutritional assessment tools, can be calculated based on the weight, height, and serum albumin levels and does not require patient cooperation. This simplicity enhances its applicability in a dementia setting. Close correlation between the GNRI and muscle function was described previously, suggesting this nutrition-related risk index could be a useful tool for identifying individuals who may benefit from targeted nutritional support and physical rehabilitation [52]. Our findings indicate that moderate or high nutritional risk, assessed by GNRI, is an independent predictor of probable sarcopenia in a multivariable binary logistic regression model adjusted for age and sex (OR 5.14, 95% CI 1.34–19.75; *p* = 0.017). The association between cut-off values of GNRI 98 and 105 with a diagnosis of sarcopenia in the overall, men, and women groups in patients with type two diabetes mellitus has been previously confirmed [53]. Apart from being a prognostic predictor for patients with different chronic diseases, a GNRI score of <104 distinguished AD patients from non-AD, and AD patients with mild cognitive impairment from the normal control group with normal cognition [47].

The evidence regarding the association between nutritional status and probable sarcopenia remains inconsistent. Some studies have found no significant relationship between malnutrition and muscle strength [54,55] while others have reported a significant association [56,57]. It is important to note, however, that different tools for assessing malnutrition were used across studies. A study of 506 patients with probable sarcopenia defined by EWGSOP2 showed no association between malnutrition as defined by GLIM and low handgrip strength in geriatric rehabilitation inpatients [58]. Probable sarcopenia and malnutrition are not consistently linked, also due to the fact that the early decline in muscle strength can be influenced by multiple factors, and not only by undernutrition. However, after the onset of muscle mass loss, which is a defining feature of confirmed sarcopenia, malnutrition starts playing a more prominent role, which accounts for the strong associations found in the literature. Confirmed sarcopenia was consistently associated with elevated malnutrition risk in multiple older adult populations—both hospitalized [59] and community-based [60,61]. According to a meta-analysis, malnutrition coexists with sarcopenia in 23% of elderly hospitalized patients [59]. Recognizing this overlap emphasizes the importance of early identification of both conditions in hospitalized patients. Simple assessments, such as handgrip strength measurement, can detect early signs and enable timely intervention in older adults at risk, particularly those with cognitive decline.

### 4.3. Polypharmacy, Comorbidity, and Muscle Function

In addition to nutritional factors, multimorbidity and polypharmacy (>5 medications) emerged as significantly different between the patients with probable sarcopenia and those without sarcopenia. Patients with probable sarcopenia were more likely to be exposed to polypharmacy and had significantly higher CCI scores. Polypharmacy was also associated with reduced HGS in our model with ACS patients, which is in line with numerous findings in the literature that highlight the detrimental effects of polypharmacy on muscle function and cognitive decline [62,63,64]. Adverse drug interactions, metabolic changes, and side effects (such as reduced physical activity, fatigue, and disturbances in the absorption of certain nutrients) can significantly contribute to the decline in muscle quality and performance in older patients. A particularly vulnerable group is patients with higher morbidity, typical of cognitive impairment and dementia, where polypharmacy is often a consequence of the treatment of multiple concomitant diseases, which was proven also by higher CCI in patients with probable sarcopenia. Our results thus highlight the need for a critical assessment of the appropriateness of drug introduction in this population, considering the balance of risks and benefits.

### 4.4. Gait Speed as a Reflection of Physical Performance

Physical performance is defined as an objectively measured whole body function related to mobility [65]. It is a multidimensional construct that reflects not only muscular capabilities but also central and peripheral nervous system function, including balance [65]. It can be assessed with various tests like gait speed, the Short Physical Performance Battery, and the Timed-Up-and-Go test, among others. Assessment of gait speed is an integral component of the diagnostic procedure for sarcopenia. According to the EWGSOP2 criteria for sarcopenia, a gait speed below 0.8 m/s defines severe sarcopenia.

Our results confirmed a strong association between probable sarcopenia and physical performance, as measured by gait speed. In line with previously published data [34], gait speed was a strong positive predictor of handgrip strength in our model. Sarcopenia and, in particular, its strength determinant, has been proposed as a potential indicator of combined cognitive and physical impairment, the presence of which may signal an increased risk for developing functional limitations and disability [32]. In our sample, we found that patients with probable sarcopenia walked significantly slower than the patients without and were more likely to fall below the clinically accepted threshold, a reliable indicator of age-related frailty and a known predictor of hospitalizations, falls, and mortality [66]. Gait speed is also recognized as an independent predictor of cognitive decline [67,68].

Taken together, these findings argue for the routine incorporation of muscle function assessments, such as handgrip strength, into standard geriatric and AD care. The simplicity and feasibility of this test make it especially useful in cognitively impaired populations where more complex evaluations may not be feasible. Nutritional support, resistance exercise, and optimization of pharmacotherapy may represent relevant targets for improving physical function and overall clinical management in patients with AD. From a clinical and caregiving perspective, nutritional and physical activity interventions should be specifically adapted to disease stage, cognitive limitations, individual nutritional needs, and physical capacity. Evidence-based recommendations for nutritional management and hydration in older adults and patients with dementia are provided in current European Society for Clinical Nutrition and Metabolism guidelines, which offer practical guidance for clinical and caregiving settings [69,70]. The window of opportunity for preserving function and independence in AD patients may be narrow; therefore, early detection and timely multimodal intervention are crucial.

### 4.5. Limitations and Future Research Directions

Several limitations should be considered when interpreting our findings. First, the cross-sectional design of the study precludes any conclusions about causality or the temporal relationship between probable sarcopenia and determinants. Second, the study population was limited to patients admitted to a single geriatric psychiatry unit, which may limit the generalizability of the findings. However, the clinic is the largest in the country, serving approximately one-quarter of the national geographic area, making our cohort broadly representative of the national population. Second, although we adjusted for age and sex using ANCOVA, residual confounding due to other factors (e.g., physical activity level, education level, dietary intake, inflammation markers, or socioeconomic status) cannot be ruled out. Third, due to the retrospective nature of data collection, some laboratory data were missing. This is expected in real-world clinical settings, where not all patients require the same analysis. Finally, despite the use of standardized clinical protocols, cognitive impairment may have affected the accuracy of physical performance measures. However, adaptations and trained staff likely minimized this bias. As this was a hospitalized cohort, the results may not be fully generalizable to community-dwelling patients with AD.

Future research should explore causal relationships between sarcopenia and cognitive decline and assess the effects of targeted multimodal interventions customized to the specific needs of patients on physical and cognitive outcomes. Longitudinal studies could also provide deeper insights into the directionality and progression of this interaction, while clinical trials are needed to test whether intervention in one domain (e.g., muscle strength) may also benefit the other (e.g., cognition, activities of daily living, …).

## 5. Conclusions

In this cross-sectional study of older hospitalized adults with ACS, probable sarcopenia according to the EWGSOP2 definition was highly prevalent and significantly associated with poorer nutritional and cognitive status, polypharmacy, and reduced physical function. Our findings highlight the need for routine screening for sarcopenia in dementia care, using feasible tools such as handgrip strength and gait speed. Patients with higher nutritional risk, lower MMSE scores, a history of polypharmacy, or slow gait speed should be prioritized for assessment of muscle strength. Given the potentially modifiable nature of sarcopenia, early identification and intervention may be of clinical relevance in the context of functional status, quality of life, and cognitive and physical decline in patients with AD.

## Figures and Tables

**Table 1:** Characteristics of the patients and results of ANCOVA.

Characteristic	Total *n* = 194	Probable Sarcopenia *n* = 137	Without Sarcopenia *n* = 57	*p*-Value	ANCOVA Adjusted for Sex and Age		
					F	*p*	Eta^2^
HGS (kg) (mean ± SD)	14.5 ± 8.4	11.0 ± 6.5	23.0 ± 5.6	<0.001	238.205	<0.001	0.556
Sex (*n*, %)				0.121			
men	56 (28.9)	44 (32.1)	12 (21.1)				
women	138 (71.1)	93 (67.9)	45 (78.9)				
Age (years) (mean ± SD)	80.9 ± 7.6	82.3 ± 7.2	77.8 ± 7.5	<0.001			
Living environment (*n*, %)				0.128			
home with family	106 (54.6)	71 (51.8)	35 (61.4)				
home alone	59 (30.4)	41 (29.9)	18 (31.6)				
residential care facility	29 (14.9)	25 (18.2)	4 (7.0)				
BMI (kg/m^2^) (mean ± SD)	25.2 ± 4.9	251 ± 4.7	25.5 ± 5.5	0.594	0.247	0.620	0.001
Risk for malnutrition (BMI < 23 kg/m^2^) (*n*, %)	66 (34.0)	45 (32.8)	21 (36.8)	0.593			
Obesity (BMI > 30 kg/m^2^) (*n*, %)	28 (14.4)	18 (13.1)	10 (17.5)	0.426			
Diabetes mellitus (*n*, %)				0.390			
no	141 (72.7)	102 (74.5)	39 (68.4)				
type 1	0 (0.0)	0 (0.0)	0 (0.0)				
type 2	53 (27.3)	35 (25.5)	18 (31.6)				
GNRI (mean ± SD)	99.0 ± 8.2	97.6 ± 8.2	102.6 ± 7.3	<0.001	10.287	0.002	0.051
GNRI categories (*n*, %)				0.006			
>98, no risk	122 (62.9)	76 (55.5)	46 (80.7)				
92–98 mild risk	38 (19.6)	30 (21.9)	8 (14.0)				
82–92 moderate risk	25 (12.9)	23 (16.8)	2 (3.5)				
<82 high risk	9 (4.6)	8 (5.8)	1 (1.8)				
CCI score (mean ± SD)	5.8 ± 1.6	6.1 ± 1.6	5.1 ± 1.2	<0.001	6.018	0.015	0.031
Number of regularly used medications (mean ± SD)	8.0 ± 2.6	8.2 ± 2.6	7.6 ± 2.7	0.134	2.236	0.136	0.012
Polypharmacy (*n*, %)	166 (85.6)	123 (89.8)	43 (75.4)	0.010			
Hemoglobin (g/L) (mean ± SD)	129.5 ± 15.3	127.7 ± 16.3	133.8 ± 11.7	0.003	5.355	0.022	0.029
Urea (mmol/L) (mean ± SD)	7.4 ± 3.9	7.8 ± 4.0	6.5 ± 3.3	0.016	2.750	0.099	0.015
Creatinine (μmol/L) (mean ± SD)	95.3 ± 73.7	100.9 ± 86.1	82.0 ± 24.1	0.513	1.348	0.247	0.007
Glucose (mmol/L) (mean ± SD)	6.0 ± 2.1	5.9 ± 2.2	6.0 ± 1.7	0.484	0.089	0.765	0.001
Cholesterol (mmol/L) (mean ± SD)	4.7 ± 1.3	4.6 ± 1.2	4.9 ± 1.3	0.089	0.749	0.388	0.005
Triglycerides (mmol/L) (mean ± SD)	1.4 ± 0.7	1.4 ± 0.7	1.4 ± 0.7	0.566	0.040	0.841	<0.001
HDL (mmol/L) (mean ± SD)	1.2 ± 0.3	1.2 ± 0.3	1.3 ± 0.3	0.015	2.058	0.153	0.012
LDL (mmol/L) (mean ± SD)	3.0 ± 1.1	2.9 ± 1.1	3.2 ± 1.2	0.338	1.416	0.236	0.009
Albumin (g/L) (mean ± SD)	39.3 ± 4.9	38.4 ± 4.9	41.4 ± 4.3	<0.001	8.905	0.003	0.045
B12 (pmol/L) (mean ± SD)	260.1 ± 118.5	262.6 ± 121.0	253.9 ± 113.0	0.817	0.781	0.378	0.005
Folic acid (nmol/L) (mean ± SD)	11.3 ± 5.2	10.9 ± 5.1	12.2 ± 5.5	0.122	0.973	0.325	0.006
Gait speed (m/s) (mean ± SD)	0.6 ± 0.3	0.5 ± 0.3	0.8 ± 0.3	<0.001	12.853	<0.001	0.066
Gait speed ≤ 0.8 m/s (*n*, %)	147 (78.6)	112 (86.2)	35 (61.4)	<0.001			
MMSE (score)	16.1 ± 6.4	15.2 ± 6.0	18.1 ± 6.7	0.005	5.284	0.023	0.027
MMSE categories (*n*, %)				0.029			
mild (≥19)	75 (38.7)	45 (32.8)	30 (52.6)				
moderate (11–18)	77 (39.7)	58 (42.3)	19 (33.3)				
severe (≤10)	42 (21.6)	34 (24.8)	8 (14.0)				

SD, standard deviation; HGS, handgrip strength; BMI, body mass index; MMSE, Mini-Mental State Examination; GNRI, Geriatric Nutritional Risk Index; CCI, Charlson comorbidity index.

**Table 2:** Association of independent predictors with handgrip strength, results of multivariable linear regression.

Variables	B	SE	95% CI for B	*p*-Value
GNRI score	0.26	0.06	0.13 to 0.38	<0.001
CCI score	−0.53	0.37	−1.26 to 0.21	0.163
Polypharmacy	−4.20	1.37	−6.90 to −1.51	0.002
Hemoglobin (g/L)	0.04	0.04	−0.03 to 0.11	0.232
Urea (mmol/L)	−0.01	0.14	−0.28 to 0.26	0.937
B12 (pmol/L)	−0.01	0.00	−0.01 to 0.00	0.202
Folic acid (nmol/L)	−0.14	0.10	−0.34 to 0.06	0.169
Gait speed (m/s)	4.12	1.57	1.02 to 7.22	0.010
MMSE score	0.17	0.08	0.02 to 0.32	0.029
Female sex	−7.30	1.09	−9.46 to −5.14	<0.001
Age	−0.09	0.07	−0.24 to 0.05	0.197

B, coefficient in regression equation; SE, standard error; CI, confidence interval; MMSE, Mini-Mental State Examination; GNRI, Geriatric Nutritional Risk Index; CCI, Charlson comorbidity index. R^2^ = 0.442 (model adjusted by sex and age).

**Table 3:** Association of independent predictors with probable sarcopenia, results of multivariable binary logistic regression.

Variables	OR (95% CI)	*p*-Value
GNRI score		
no risk (>98)	1.00 (reference)	
mild risk (92–98)	1.99 (0.75 to 5.29)	0.170
moderate or high risk (<92) *	5.14 (1.34 to 19.75)	0.017
CCI score (per 1 unit increase)	1.29 (0.91 to 1.84)	0.154
Polypharmacy	2.45 (0.93 to 6.46)	0.071
Hemoglobin (M < 130 g/L or F < 120 g/L)	1.45 (0.52 to 4.03)	0.471
Urea > 7.9 mmol/L	0.80 (0.32 to 1.99)	0.626
B12 (pmol/L)		
<138	1.00 (reference)	
138–652	0.89 (0.28 to 2.85)	0.847
>652	1.51 (0.26 to 8.84)	0.649
Folic acid (nmol/L)		
<7	1.00 (reference)	
7–46.4	1.47 (0.56 to 3.83)	0.430
>46.4	1.28 (0.10 to 16.75)	0.852
Gait speed ≤ 0.8 m/s	3.13 (1.30 to 7.52)	0.011
MMSE score		
mild (≥19)	1.00 (reference)	
moderate (11–18)	1.95 (0.86 to 4.44)	0.111
severe (≤10)	1.97 (0.70 to 5.56)	0.199
Female sex	0.62 (0.26 to 1.48)	0.284
Age (per 10 years increase)	1.65 (0.97 to 2.82)	0.067

OR, odds ratio; CI, confidence interval; MMSE, Mini-Mental State Examination; GNRI, Geriatric Nutritional Risk Index; CCI, Charlson comorbidity index. * Because of the limited sample size in the high nutritional risk category (n = 9), moderate and high nutritional risk were merged into a single category for the logistic regression analysis. Nagelkerke R^2^ = 0.330 (model adjusted by sex and age).

## Data Availability

The raw data supporting the conclusions of this article will be made available by the authors on request.

## Abbreviations

The following abbreviations are used in this manuscript:

ACS	Alzheimer’s clinical syndrome
AD	Alzheimer’s disease
EWGSOP2	European Working Group on Sarcopenia in Older People, updated 2018
HGS	Handgrip strength
NIA-AA	National Institute on Aging—Alzheimer’s Association
BMI	Body mass index
MMSE	Mini-Mental State Examination
GNRI	Geriatric Nutritional Risk Index
CCI	Charlson comorbidity index
